# Editorial: Targeting Pancreatic Cancer: Strategies and Hopes

**DOI:** 10.3389/fonc.2022.873682

**Published:** 2022-03-31

**Authors:** Kanjoormana Aryan Manu, Kuzhuvelil B. Harikumar, Takatsugu Ishimoto

**Affiliations:** ^1^ Department of Immunology, Amala Cancer Research Centre, Thrissur, India; ^2^ Cancer Research Program, Rajiv Gandhi Centre for Biotechnology (RGCB), Thiruvananthapuram, India; ^3^ Takatsugu Ishimoto, International Research Centre for Medical Sciences, Kumamoto University, Kumamoto, Japan

**Keywords:** pancreatic cancer, therapeutics, tumor microenvironment, metastasis, nomograms

Last couple of decades showed remarkable progress in the area of cancer research which improved the quality of diagnosis and treatment, resulted in substantial increase in cancer recovery rate. But the scenario is different for Pancreatic cancers, where the percentage of mortality is still as high as 95% which failed to decline. A study in 28 European countries, projected that pancreatic cancer will surpass breast cancer as the third leading cause of cancer death by 2025 (1). Surgical resection is the only possible treatment for pancreatic cancers which can be followed by adjuvant chemo therapies. To date there is no targeted therapy available for pancreatic cancers and it is the need of the hour to be more focused on pancreatic cancer and compile recent research outputs which help us to design our future directions. Our Research Topic was designed to look at the current and future strategies of pancreatic cancer treatments which may give hope to the patients. This Research Topic consists of 13 articles including 8 Original research articles, 4 reviews and 1 case report.

Development of early diagnosis and use of an effective personalised approach is one of the promising strategies to improve therapeutic outcome in pancreatic cancer patients. Miyabashi et al. have reviewed the promising details of combining genome-based medicine with drug screening based on personalized models which may direct to the use of precision medicine for pancreatic cancer. The liquid biopsy and use of three-dimensional organoid culture or patient-derived xenografts platforms also have been discussed in the review article.

Takahashi et al. have described the development of a novel method to detect pancreatic tumors using a tumor-specific enzyme-activatable fluorescence probe which helps rapid and real-time visualization of pancreatic cancer through the enzymatic activities of cancer tissues. This novel technique can accurately identify the extent of the tumor before and during surgery.

Use of bioinformatics tools and machine learning algorithms may be useful for predicting pancreatic cancer patient prognosis. Li et al. discussed the use of the ‘Extreme gradient boosting classifier’ (XGBoost) to predict CD8+ tumor-infiltrating lymphocyte expression levels in patients with pancreatic ductal adenocarcinoma (PDAC) using CT radiomic features. Tan et al. constructed a novel four genes signature to predict the prognosis of Stages III and IV PDAC patients by applying WGCNA and CIBERSORT algorithm scoring to transcriptome data different from traditional methods of filtrating for differential genes in cancer and healthy tissues. The findings may provide reference to predict survival and be beneficial for individualized management of advanced PDAC patients.

Pappalardo et al. published a review in this special issue on the current treatment scenario and new potential therapeutic approaches in early stage PDAC, from both preclinical and clinical point of view. Lewis et al. published their new finding of a rabbit monoclonal antibody specific for human alternatively spliced tissue factor (asTF) and evaluated its binding characteristics and assessed its *in vivo* properties. Peng et al. evaluated the efficacy of hepatic artery infusion (HAI) of floxuridine (FUDR) in combination with systemic chemotherapy in patients with pancreatic cancer liver metastases (PCLM).

YAP1 is a transcriptional coactivator and a downstream effector of Hippo signaling (2). It has been reported to process a significant role in development of PDAC and progression (3). Gao et al. did a thorough study and showed that both YAP1-1 and YAP1-2 isoforms are important mediators in the EMT process of pancreatic cancer. Hayashi et al. reviewed the role of YAP in PDACs and summarised the biological significance of a dysregulated Hippo signaling pathway PDACs.

Tumor microenvironment consists cancer associated fibroblasts, CD4^+^ T cells and myeloid cells, which are linked and can influence each other which contribute to cancer cell plasticity, invasiveness, metastasis, chemo-resistance, immunotherapy-resistance and radiotherapy-resistance (4). In a review, Du et al. characterized prevailing population of stromal cells in Pancreatic Ductal Adenocarcinoma tumors and how it interact with the other components of tumor microenvironment leading to tumor progression and described how the re-programming of tumor microenvironment improve treatment outcome for pancreatic cancer patients.

Lewis et al. described the pre-clinical evaluation of a humanized antibody targeting alternatively spliced tissue factor. It shows significant activity as a single agent and RNAseq analysis of tumors treated with this monoclonal antibody showed a significant decrease in the expression of genes associated with focal adhesion and cell cycle progression.

Identification of new targetable genes or proteins are important in developing novel therapeutics. Lack of targeted therapy is the major setback for pancreatic cancer treatment. In this Research Topic, Chen et al. reported identification of a novel miRNA hsa-mir-4772 and two novel genes (COL12A1 and COL5A2) associated with pancreatic cancer, which can be used as prognostic factors and therapeutic targets for pancreatic cancer.

Raoul et al. published a case study of a young woman with long term stabilization of a G2 metastatic pancreatic NET that, after pregnancy, suddenly progressed into one single liver metastasis corresponding to a transformation into G3 large-cell neuroendocrine cancer. Authors are raising a question for future research about the role of temozolomide which used in this patient in combination with capecitabine during G2 metastatic pancreatic NET treatment.

More focus has been required in pancreatic cancer research in order to develop early diagnosis methodologies and targeted medicines to tackle this disease and reduce mortality. Development of new liquid biopsy techniques give hopes for the people to get the disease diagnosed early which certainly improves the survival chance of patients. Use of personalised therapies, developing neoadjuvant treatments, combination therapies and identifying new molecular targets in cells and tumor microenvironment are also highly promising to give hope for patients. Parallel developments in the area of bioinformatics and new deep learning, data science and machine learning approaches and its application in pancreatic cancer research and therapy also gives ample hope for us. This Research Topic not only compiles research outputs in the field but also gives a real hope that ‘the light of success’ is not very far.

## Author Contributions

KM has written the editorial. HB and TI reviewed and edited the editorial. All authors contributed to the article and approved the submitted version.

## Conflict of Interest

The authors declare that the research was conducted in the absence of any commercial or financial relationships that could be construed as a potential conflict of interest.

## Publisher’s Note

All claims expressed in this article are solely those of the authors and do not necessarily represent those of their affiliated organizations, or those of the publisher, the editors and the reviewers. Any product that may be evaluated in this article, or claim that may be made by its manufacturer, is not guaranteed or endorsed by the publisher.
